# Supplementary material for: Synthesis of Halogenated 1,5-Diarylimidazoles and Their Inhibitory Effects on LPS-Induced PGE2 Production in RAW 264.7 Cells
Source: Molecules. 2021 Oct 9;26(20):6093. doi: 10.3390/molecules26206093 (PMC8538130; doi:10.3390/molecules26206093)
Supplement: Supplementary file 1 [file molecules-26-06093-s001.zip › molecules-1406342-supplementary.pdf]

Supplementary Data

Synthesis

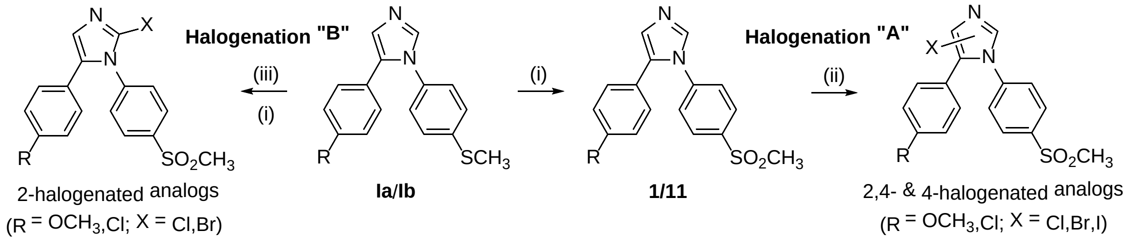

Scheme 1. Synthetic pathways and conditions of 5-aryl-1-(4-methylsulfonylphenyl)imidazoles with halogen(s) on the imidazole ring : (i) MCPBA, DCM (ii) NCS/NBS/NIS, $CHCl_3$ (iii) NCS/NBS, LiHMDS, THF

General procedure for 5-ary-1-(4-methylthiophenyl)imidazoles: To the solution of imine (4 mmol) in MeOH and DME (20 mL, *v/v*=1/2) was added anhydrous potassium carbonate (1.66 g, 12 mmol) and tosylmethyl isocyanide (0.94 g, 4.8 mmol). The reaction mixture was refluxed for 1 day. The solvent was removed, and the residue was extracted with DCM. The organic layer was washed with brine, dried over magnesium sulfate and concentrated. The residue was purified by silica gel column chromatography with hexane-EtOAc mixture as the eluent. **5-(4-methoxyphenyl)-1-(4-methylthiophenyl)imidazole (Ia).** The product was obtained as a yellow solid with a yield of 10%; ^{1}H-NMR (300 MHz, $CDCl_3$) δ 7.65 (s, 1H, 2-H), 7.24 (d, J = 8.6 Hz, 2H, Ar-H), 7.18 (s, 1H, 4-H), 7.08 (m, 4H, Ar-H), 6.81 (d, J = 8.8 Hz, 2H, Ar-H), 3.79 (s, 3H, OCH_3) , 2.50 (s, 3H, SCH_3). **5-(4-chorophenyl)-1-(4-methylthiophenyl)imidazole (Ib)** The product was obtained as a yellow solid with a yield of 49%; ^{1}H-NMR (300 MHz, $CDCl_3$) δ 7.67 (d, J = 1.0 Hz, 1H, 2-H), 7.30-7.20 (m, 5H, Ar-H, 4-H), 7.07 (m, 4H, Ar-H), 2.51 (s, 3H, CH_3).

General procedure for 5-aryl-1-(4-methylsulfonylphenyl)imidazoles: To the solution of intermediate **I** (1 mmol) in DCM (10 mL) was added, at 0°C, 3-chloroperbenzoic acid (0.56 g, 2.5 mmol). The mixture was stirred for 2 h, added more DCM, washed with aqueous $Na_2S_2O_3$, $NaHCO_3$ and brine and dried over magnesium sulfate and concentrated. The residue was purified by silica gel column chromatography with hexane-EtOAc mixture as the eluent. **1-(4-methylsulfonylphenyl)-5-(4-methoxyphenyl)imidazole (1).** The product was obtained as a white solid with a yield of 84%; ^{1}H-NMR (300 MHz, $CDCl_3$) δ 7.95 (d, J = 8.6 Hz, 2H, Ar-H), 7.74 (s, 1H, 2-H), 7.38 (d, J = 8.6 Hz, 2H, Ar-H), 7.22 (s, 1H, 4-H), 7.05 (d, J = 8.8 Hz, 2H, Ar-H), 6.84 (d, J = 8.8 Hz, 2H, Ar-H), 3.80 (s, 3H, OCH_3) , 3.08 (s, 3H, SO_2CH_3); ESIMS: *m/z* $[M+H]^+$ 329.1 ; **5-(4-chorophenyl)-1-(4-methylsulfonylphenyl)imidazole (11).** The product was obtained as a yellow solid with a yield of 87%; ^{1}H-NMR (300 MHz, $CDCl_3$) δ 8.02 (d, J = 8.6 Hz, 2H, Ar-H), 7.78 (s, 1H, 2-H), 7.30 (m, 5H, Ar-H, 4-H), 7.05 (d, J = 8.5 Hz, 2H, Ar-H), 3.11 (s, 3H, CH_3).

General procedure for halogenation of 5-aryl-1-(4-methylsulfonylphenyl)imidazoles: To the solution of compound **1/11** (0.5 mmol) in $CHCl_3$ (4 mL) was added NCS/NBS/NIS (0.75 mmol). The mixture was refluxed for 5 h, extracted with DCM, washed with aqueous $NaHSO_3$ and brine and dried over magnesium sulfate and concentrated. The residue was purified by silica gel column chromatography to give 4-halo and 2,4-dihalo imidazoles. Different from chlorination, 2-bromo imidazole products were separated in some bromination reactions. While iodination with NIS in CH_3CN always afforded 2-, 4- and 2,4-iodo imidazole products.

2,4-dichloro)-5-(4-methoxyphenyl)-1-(4-methylsulfonylphenylimidazole (2). The product was obtained as a white solid with a yield of 15%; ^{1}H-NMR (300 MHz, $CDCl_3$) δ 8.00 (d, J = 8.7 Hz, 2H, Ar-H), 7.37 (d, J = 8.7 Hz, 2H, Ar-H), 7.03 (d, J = 8.9 Hz, 2H, Ar-H), 6.80 (d, J = 8.9 Hz, 2H, Ar-H), 3.79 (s, 3H, OCH_3) , 3.11 (s, 3H, SO_2CH_3). **4-chloro-1-(4-methylsulfonylphenyl)-5-(4-methoxyphenyl)imidazole (3).** The product was obtained as a white solid with a yield of 41%; ^{1}H-NMR (300 MHz, $CDCl_3$) δ 7.95 (d, J = 8.6 Hz, 2H, Ar-H), 7.65 (s, 1H, 2-H), 7.32 (d, J = 8.6 Hz, 2H, Ar-H), 7.11 (d, J = 8.8 Hz, 2H, Ar-H), 6.88 (d, J = 8.8 Hz, 2H, Ar-H), 3.82 (s, 3H, OCH_3) , 3.09 (s, 3H, SO_2CH_3); ^{13}C-NMR (150 MHz, $CDCl_3$) δ 159.9, 140.6, 135.0, 129.1, 129.0, 127.1, 125.7, 118.7, 114.4, 55.3, 44.4; HRMS (EI) m/z Calcd for C17H15ClN2O3S [M]+ 362.0492 Found 362.0493.

2,4-dibromo-5-(4-methoxyphenyl)-1-(4-methylsulfonylphenyl)imidazole (5). The product was obtained as a white solid with a yield of 15%; ^{1}H-NMR (300 MHz, $CDCl_3$) δ 7.98 (d, J = 8.6 Hz, 2H, Ar-H), 7.36 (d, J = 8.7 Hz, 2H, Ar-H), 7.04 (d, J

= 8.9 Hz, 2H, Ar-H), 6.80 (d, J = 8.9 Hz, 2H, Ar-H), 3.79 (s, 3H, OCH_3) , 3.11 (s, 3H, SO_2CH_3). **4-bromo-5-(4-methoxyphenyl)-1-(4-methylsulfonylphenyl)imidazole (6)** The product was obtained as a white solid with a yield of 68%; ^{1}H-NMR (300 MHz, $CDCl_3$) δ 7.95 (d, J = 8.7 Hz, 2H, Ar-H), 7.70 (s, 1H, 2-H), 7.31 (d, J = 8.7 Hz, 2H, Ar-H), 7.15 (d, J = 8.8 Hz, 2H, Ar-H), 6.85 (d, J = 8.8 Hz, 2H, Ar-H), 3.82 (s, 3H, OCH_3) , 3.08 (s, 3H, SO_2CH_3).

2,4-diiodo-5-(4-methoxyphenyl)-1-(4-methylsulfonylphenyl)imidazole (8). The product was obtained as a yellow solid with a yield of 5%; ^{1}H-NMR (300 MHz, $CDCl_3$) δ 7.91 (d, J = 8.7 Hz, 2H, Ar-H), 7.25 (d, J = 8.7 Hz, 2H, Ar-H), 6.95 (d, J = 8.9 Hz, 2H, Ar-H), 6.72 (d, J = 8.9 Hz, 2H, Ar-H), 3.71 (s, 3H, OCH_3) , 3.04 (s, 3H, SO_2CH_3). **4-iodo-5-(4-methoxyphenyl)-1-(4-methylsulfonylphenyl) imidazole (9).** The product was obtained as a yellow solid with a yield of 23%; ^{1}H-NMR (300 MHz, $CDCl_3$) δ 7.94 (d, J = 8.7 Hz, 2H, Ar-H), 7.76 (s, 1H, 2-H), 7.28 (d, J = 8.7 Hz, 2H, Ar-H), 7.12 (d, J = 8.9 Hz, 2H, Ar-H), 6.88 (d, J = 8.9 Hz, 2H, Ar-H), 3.83 (s, 3H, OCH_3) , 3.08 (s, 3H, SO_2CH_3) ; ESIMS: *m/z* $[M+H]^+$ 455.0 ; **2-iodo-5-(4-methoxyphenyl)-1-(4-methylsulfonylphenyl)imidazole (10).** The product was obtained as a yellow solid with a yield of 6%; ^{1}H-NMR (300 MHz, $CDCl_3$) δ 7.95 (d, J = 8.7 Hz, 2H, Ar-H), 7.35 (d, J = 8.7 Hz, 2H, Ar-H), 7.18 (s, 1H, 4-H), 6.86 (d, J = 8.9 Hz, 2H, Ar-H), 6.69 (d, J = 8.9 Hz, 2H, Ar-H), 3.70 (s, 3H, OCH_3) , 3.06 (s, 3H, SO_2CH_3) ; ESIMS: *m/z* $[M+H]^+$ 455.0.

2,4-dichloro-5-(4-chorophenyl)-1-(4-methylsulfonylphenyl)imidazole (12). The product was obtained as a yellow solid with a yield of 24%; ^{1}H-NMR (300 MHz, $CDCl_3$) δ 8.02 (d, J = 8.7 Hz, 2H, Ar-H), 7.40 (d, J = 8.7 Hz, 2H, Ar-H), 7.28 (d, J = 8.7 Hz, 2H, Ar-H), 7.06 (d, J = 8.7 Hz, 2H, Ar-H), 3.12 (s, 3H, CH_3). **4-chloro-5-(4-chorophenyl)-1-(4-methylsulfonylphenyl)imidazole (13).** The product was obtained as a yellow solid with a yield of 20%; ^{1}H-NMR (300 MHz, $CDCl_3$) δ 8.00 (d, J = 8.6 Hz, 2H, Ar-H), 7.67 (s, 1H, 2-H), 7.32 (m, 4H, Ar-H), 7.13 (d, J = 8.5 Hz, 2H, Ar-H), 3.10 (s, 3H, CH_3); ^{13}C-NMR (150 MHz, $CDCl_3$) δ140.8, 140.1, 135.9, 135.2, 131.0, 129.3, 129.2, 125.8, 124.9, 55.1, 49.2, 44.4; HRMS (EI) m/z Calcd for C16H12Cl2N2O2S [M]+ 365.9997 Found 365.9994.

2,4-dibromo-5-(4-chorophenyl)-1-(4-methylsulfonylphenyl)imidazole (15). The product was obtained as a yellow solid with a yield of 15%; ^{1}H-NMR (300 MHz, $CDCl_3$) δ 8.01 (d, J = 8.7 Hz, 2H, Ar-H), 7.37 (d, J = 8.7 Hz, 2H, Ar-H), 7.27 (d, J = 8.7 Hz, 2H, Ar-H), 7.07 (d, J = 8.7 Hz, 2H, Ar-H), 3.12 (s, 3H, CH_3). **4-bromo-5-(4-chorophenyl)-1-(4-methylsulfonylphenyl)imidazole (16).** The product was obtained as a yellow solid with a yield of 50%; ^{1}H-NMR (300 MHz, $CDCl_3$) δ 7.98 (d, J = 8.7 Hz, 2H, Ar-H), 7.72 (s, 1H, 2-H), 7.33 (m, 4H, Ar-H), 7.16 (d, J = 8.6 Hz, 2H, Ar-H), 3.10 (s, 3H, CH_3); ^{13}C-NMR (150 MHz, $CDCl_3$) δ 140.5, 140.3, 137.1, 135.0, 131.2, 129.2, 129.2, 128.6, 125.8, 125.7, 117.6, 44.4; HRMS (EI) m/z Calcd for C16H12BrClN2O2S [M]+ 409.9491 Found 409.9490. **2-bromo-5-(4-chorophenyl)-1-(4-methylsulfonylphenyl)-imidazole (17).** The product was obtained as a white solid with a yield of 11%; ^{1}H-NMR (300 MHz, $CDCl_3$) δ 8.05 (d, J = 8.7 Hz, 2H, Ar-H), 7.43 (d, J = 8.7 Hz, 2H, Ar-H), 7.26 (s, 1H, 4-H), 7.24 (d, J = 8.7 Hz, 2H, Ar-H), 6.96 (d, J = 8.7 Hz, 2H, Ar-H), 3.14 (s, 3H, CH_3).

5-(4-chorophenyl)-2,4-diiodo-1-(4-methylsulfonylphenyl)imidazole (18). The product was obtained as a yellow solid with a yield of 14%; ^{1}H-NMR (300 MHz, $CDCl_3$) δ 7.94 (d, J = 8.4 Hz, 2H, Ar-H), 7.28 (d, J = 8.4 Hz, 2H, Ar-H), 7.20 (d, J = 8.4 Hz, 2H, Ar-H), 6.98 (d, J = 8.4 Hz, 2H, Ar-H), 3.05 (s, 3H, CH_3). **5-(4-chorophenyl)-4-iodo-1-(4-methylsulfonylphenyl)imidazole (19).** The product was obtained as a yellow solid with a yield of 42%; ^{1}H-NMR (300 MHz, $CDCl_3$) δ 7.89 (d, J = 8.4 Hz, 2H, Ar-H), 7.71 (s, 1H, 2-H), 7.26 (d, J = 8.4 Hz, 2H, Ar-H), 7.22 (d, J = 8.4 Hz, 2H, Ar-H), 7.08 (d, J = 8.4 Hz, 2H, Ar-H), 3.10 (s, 3H, CH_3). **5-(4-chorophenyl)-2-iodo-1-(4-methylsulfonylphenyl)imidazole (20).** The product was obtained as a white solid with a yield of 24%; ^{1}H-NMR (300 MHz, $CDCl_3$) δ 8.05 (d, J = 8.5 Hz, 2H, Ar-H), 7.43 (d, J = 8.5 Hz, 2H, Ar-H), 7.32 (s, 1H, 4-H), 7.22 (d, J = 8.5 Hz, 2H, Ar-H), 6.95 (d, J = 8.5 Hz, 2H, Ar-H), 3.15 (s, 3H, CH_3) ; ESIMS: *m/z* $[M+H]^+$ 458.9.

Alternative procedure for synthesis of 2-chloro- and 2-bromo-5-aryl-1-(4-methylsulfonylphenyl)imidazoles: To the solution of 5-aryl-1-(4-methylthiophenyl)imidazoles (**Ia** and **Ib**, 0.4 mmol) in THF (3 mL) was added LiHMDS (1 M in THF, 1.2 mL) dropwise at -20°C. The mixture was stirred for 0.5 h, then solution of NCS or NBS (1.6 mmol) in THF (3 mL) was added. The reaction mixture was stirred for 0.5 h at -20°C and 6 h at room temperature. Saturated aqueous NH_4Cl was added to the mixture, extracted with ethyl acetate. The organic layer was washed with aqueous $NaHSO_3$ and brine, dried over magnesium sulfate, and concentrated under vacuum. Following oxidation of crude 2-chloro- and 2-bromo-5-aryl-1-(4-methylthiophenyl)imidazole with *m*CPBA followed by silica gel column chromatography yielded pure 2-chloro- and 2-bromo-5-aryl-1-(4-methylsulfonylphenyl)imidazoles.

2-chloro-5-(4-methoxyphenyl)-1-(4-methylsulfonylphenyl)imidazole (4). The product was obtained as a white solid with a yield of 14%; ^{1}H-NMR (300 MHz, $CDCl_3$) δ 8.01 (d, J = 8.5 Hz, 2H, Ar-H), 7.41 (d, J = 8.5 Hz, 2H, Ar-H), 7.10 (s, 1H, 4-H), 6.95 (d, J = 8.8 Hz, 2H, Ar-H), 6.78 (d, J = 8.8 Hz, 2H, Ar-H), 3.78 (s, 3H, OCH_3) , 3.12 (s, 3H, SO_2CH_3). **2-bromo-5-(4-methoxyphenyl)-1-(4-methylsulfonylphenyl)imidazole (7).** The product was obtained as a white solid with a yield of 12%; ^{1}H-NMR (300 MHz, $CDCl_3$) δ 7.96 (d, J = 8.7 Hz, 2H, Ar-H), 7.64 (s, 1H, 4-H), 7.32 (d, J = 8.7 Hz, 2H, Ar-H), 7.12 (d, J = 8.8 Hz, 2H, Ar-H), 6.88 (d, J = 8.8 Hz, 2H, Ar-H), 3.82 (s, 3H, OCH_3) , 3.09 (s, 3H, SO_2CH_3); ^{13}C-NMR (150 MHz, $CDCl_3$) δ 159.9, 140.7, 140.1, 136.4, 131.3, 129.6, 129.0, 125.7, 119.3, 116.9, 114.3, 55.3, 44.4; HRMS (EI) m/z Calcd for C17H15BrN2O3S [M]+ 405.9987 Found 405.9988.

2-chloro-5-(4-chorophenyl)-1-(4-methylsulfonylphenyl)imidazole (14). The product was obtained as a white solid with a yield of 12%; ^{1}H-NMR (300 MHz, $CDCl_3$) δ 8.05 (d, J = 8.7 Hz, 2H, Ar-H), 7.42 (d, J = 8.7 Hz, 2H, Ar-H), 7.22 (d, J = 8.6 Hz, 2H, Ar-H), 7.19 (s, 1H, 4-H), 6.96 (d, J = 8.6 Hz, 2H, Ar-H), 3.13 (s, 3H, CH_3).

Biology

MTT assay

Effects of 9 – 20 and Celecoxib on cell viability in Raw 264.7 cells

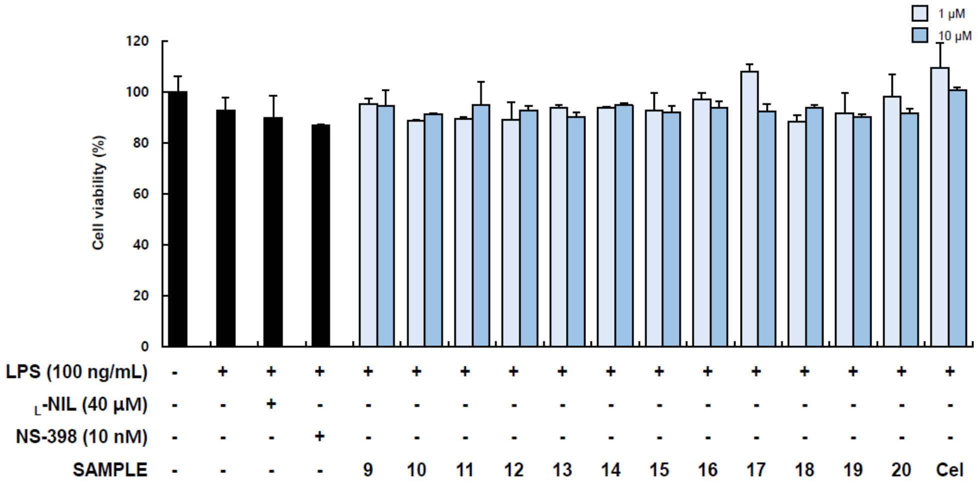

Effects of 9 – 20 and Celecoxib on cell viability in Raw 264.7 cells

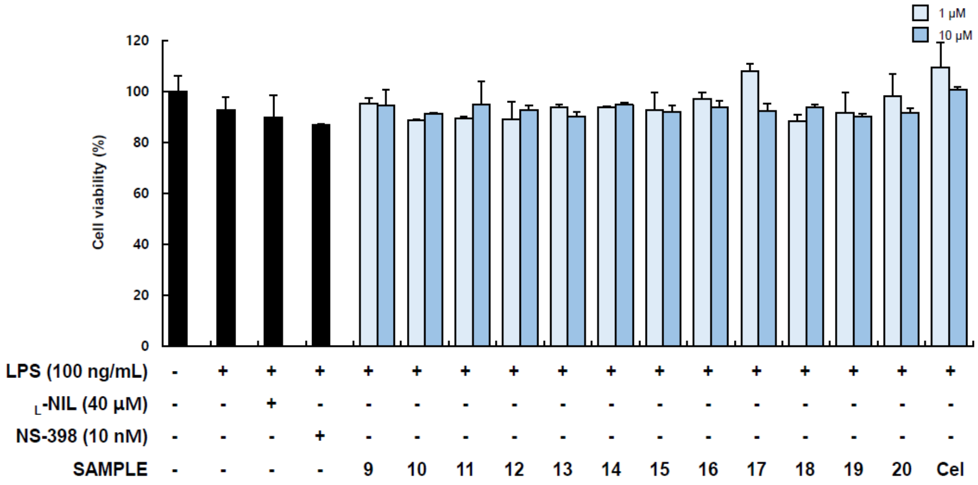

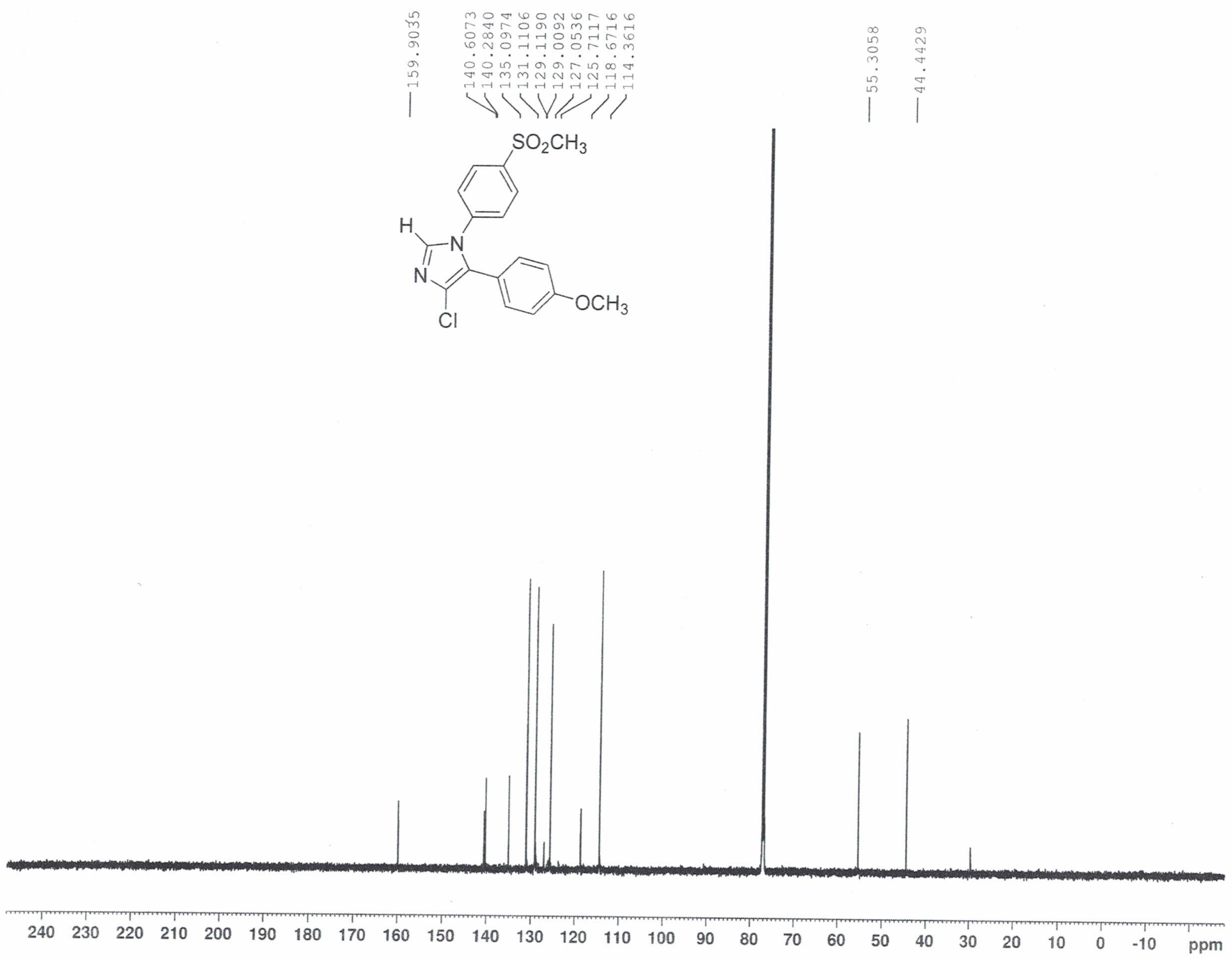

BRUKER

Current Data Parameters

NAME	210708
EXPNO	12
PROCNO	1

F2 - Acquisition Parameters

Date_	20210708
Time	12.19 h
INSTRUM	Avance
PROBHD	Z114607_0856 (
PULPROG	zgpg30
TD	65536
SOLVENT	CDCl3
NS	1024
DS	4
SWH	41666.668 Hz
FIDRES	1.271566 Hz
AQ	0.7864320 sec
RG	101
DW	12.000 use
DE	6.50 use
TE	298.0 K
D1	2.00000000 sec
D11	0.03000000 sec
TD0	1
SFO1	150.9445556 MHz
NUC1	13C
P0	3.90 use
P1	11.70 use
PLW1	80.00000000 W
SFO2	600.2324009 MHz
NUC2	1H
CPDPRG[2	waltz65
PCPD2	70.00 use
PLW2	33.82600021 W
PLW12	0.69032001 W
PLW13	0.34722999 W

F2 - Processing parameters

SI	32768
SF	150.9279535 MHz
WDW	EM
SSB	0
[illegible]	[illegible]

7

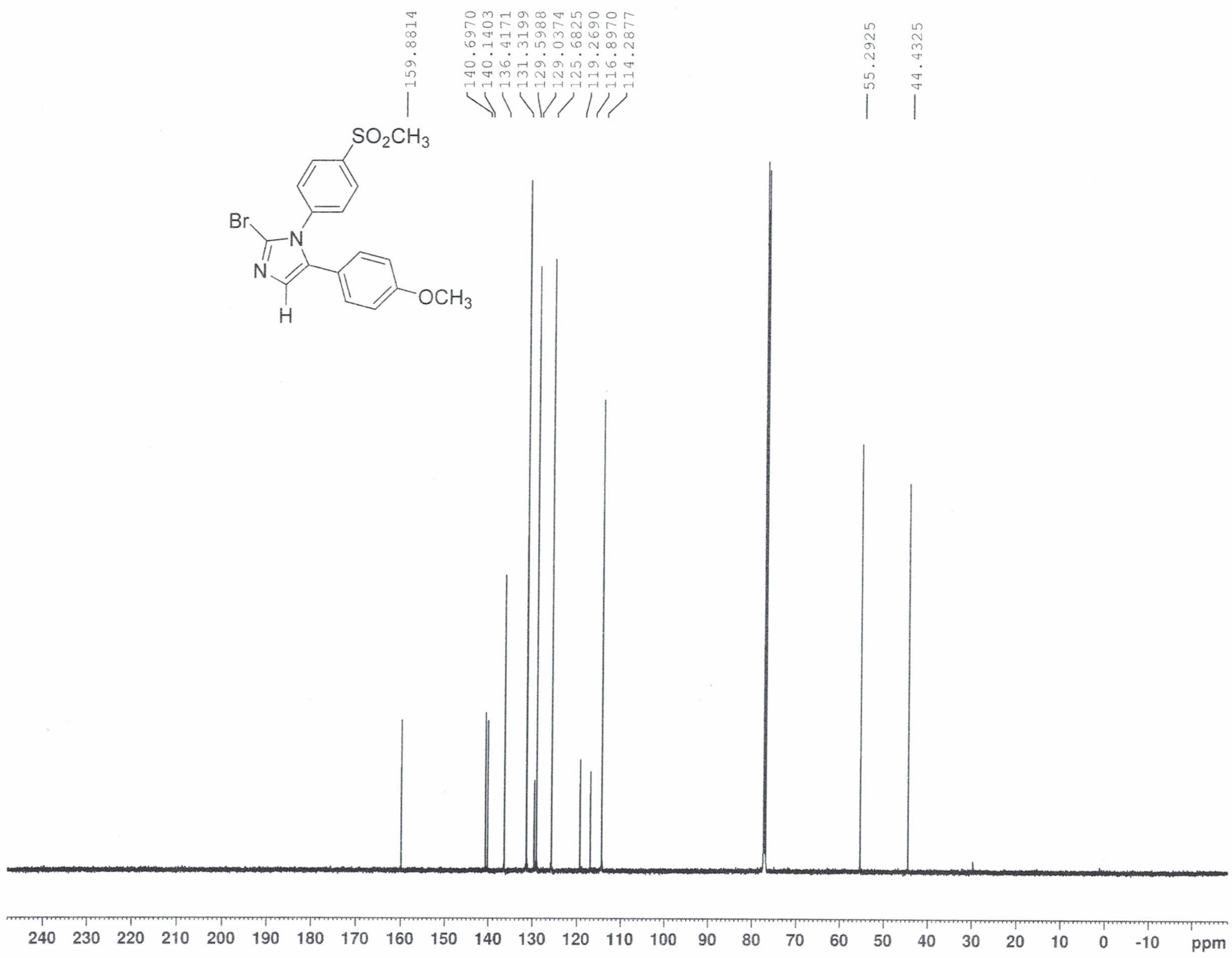

BRUKER

```
Current Data Parameters
NAME              210708
EXPNO                 21
PROCNO                 1

F2 - Acquisition Parameters
Date_           20210708
Time               11.25 h
INSTRUM           Avance
PROBHD   Z114607_0856 (
PULPROG          zgpg30
TD                 65536
SOLVENT            CDCl3
NS                  1024
DS                     4
SWH           41666.668 Hz
FIDRES         1.271566 Hz
AQ            0.7864320 sec
RG                   101
DW                12.000 use
DE                  6.50 use
TE                 298.0 K
D1            2.00000000 sec
D11           0.03000000 sec
TD0                    1
SFO1         150.9445556 MHz
NUC1                 13C
P0                  3.90 use
P1                 11.70 use
PLW1         80.00000000 W
SFO2         600.2324009 MHz
NUC2                  1H
CPDPRG[2         waltz65
PCPD2              70.00 use
PLW2         33.82600021 W
PLW12         0.69032001 W
PLW13         0.34722999 W

F2 - Processing parameters
SI                 32768
SF           150.9279535 MHz
WDW                   EM
SSB                    0
[illegible]
```

#13

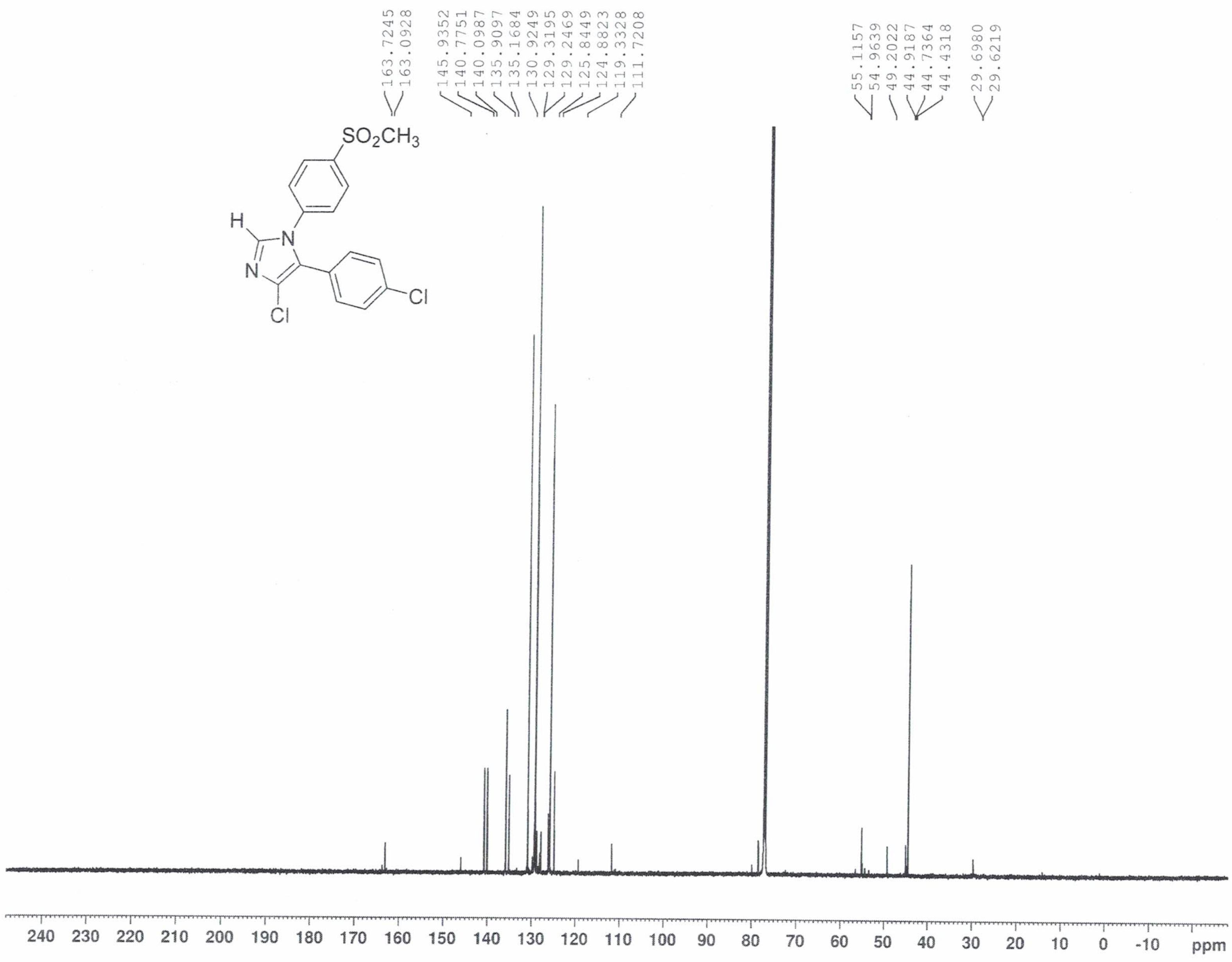


```
Current Data Parameters
NAME              210706
EXPNO                 31
PROCNO                 1

F2 - Acquisition Parameters
Date_           20210707
Time                5.33 h
INSTRUM           Avance
PROBHD    Z114607_0856 (
PULPROG          zgpg30
TD                 65536
SOLVENT            CDCl3
NS                  5120
DS                     4
SWH           41666.668 Hz
FIDRES         1.271566 Hz
AQ            0.7864320 sec
RG                   101
DW                12.000 use
DE                  6.50 use
TE                 298.0 K
D1            2.00000000 sec
D11           0.03000000 sec
TD0                    1
SFO1         150.9445556 MHz
NUC1                 13C
P0                  3.90 use
P1                 11.70 use
PLW1         80.00000000 W
SFO2         600.2324009 MHz
NUC2                  1H
CPDPRG[2          waltz65
PCPD2              70.00 use
PLW2         33.82600021 W
PLW12         0.69032001 W
PLW13         0.34722999 W

F2 - Processing parameters
SI                 32768
SF           150.9279535 MHz
WDW                   EM
SSB                    0
```

#16

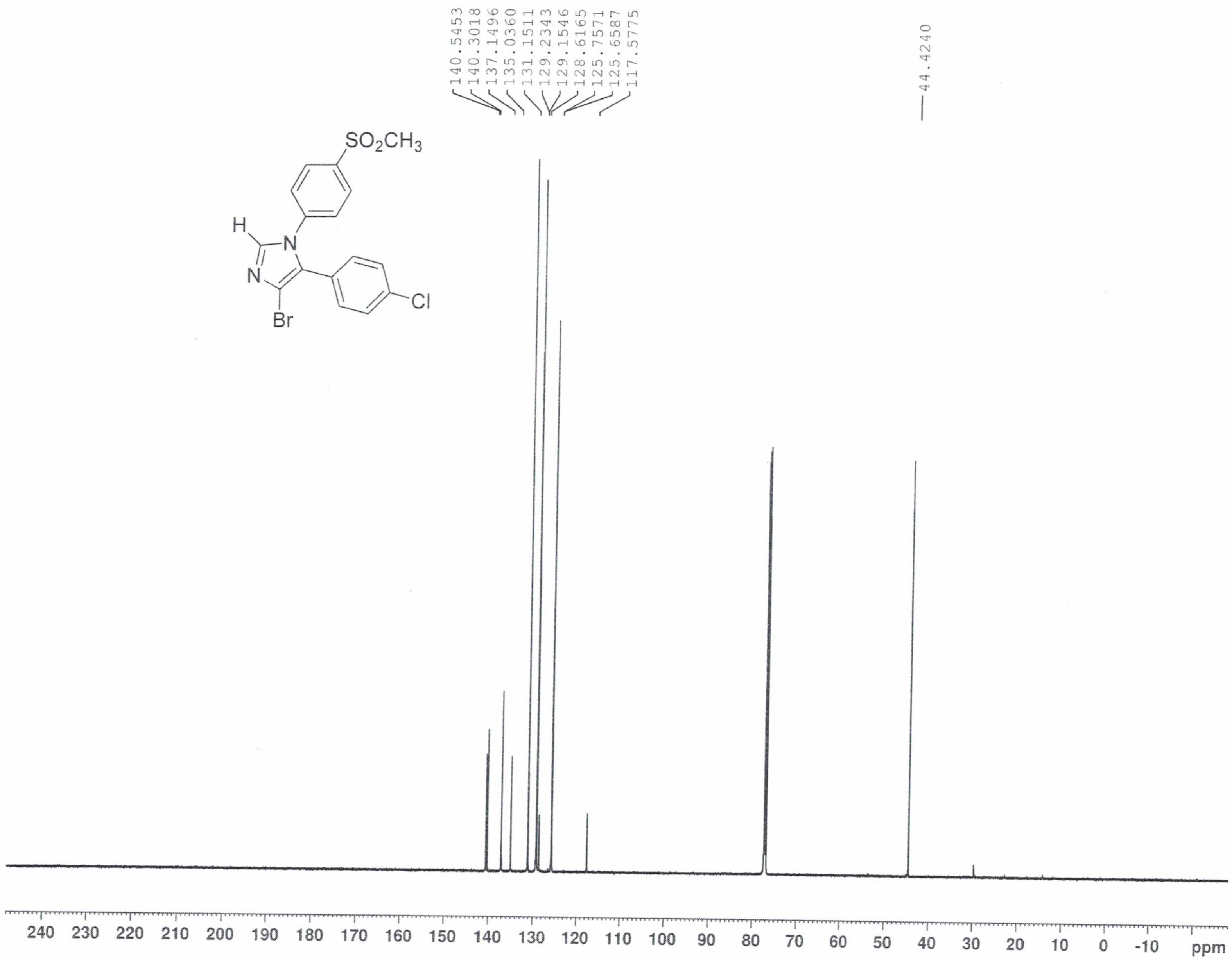

BRUKER

Current Data Parameters	
NAME	210706
EXPNO	41
PROCNO	1

F2 - Acquisition Parameters	
Date_	20210707
Time	9.56 h
INSTRUM	Avance
PROBHD	Z114607_0856 (
PULPROG	zgpg30
TD	65536
SOLVENT	CDCl3
NS	5120
DS	4
SWH	41666.668 Hz
FIDRES	1.271566 Hz
AQ	0.7864320 sec
RG	101
DW	12.000 use
DE	6.50 use
TE	298.0 K
D1	2.00000000 sec
D11	0.03000000 sec
TD0	1
SFO1	150.9445556 MHz
NUC1	13C
P0	3.90 use
P1	11.70 use
PLW1	80.00000000 W
SFO2	600.2324009 MHz
NUC2	1H
CPDPRG[2	waltz65
PCPD2	70.00 use
PLW2	33.82600021 W
PLW12	0.69032001 W
PLW13	0.34722999 W

F2 - Processing parameters	
SI	32768
SF	150.9279535 MHz
WDW	EM
SSB	0
[illegible]	[illegible]

ple 1 (TuneSampleID) of Sample 1 (210709).wiff (Turbo Spray)

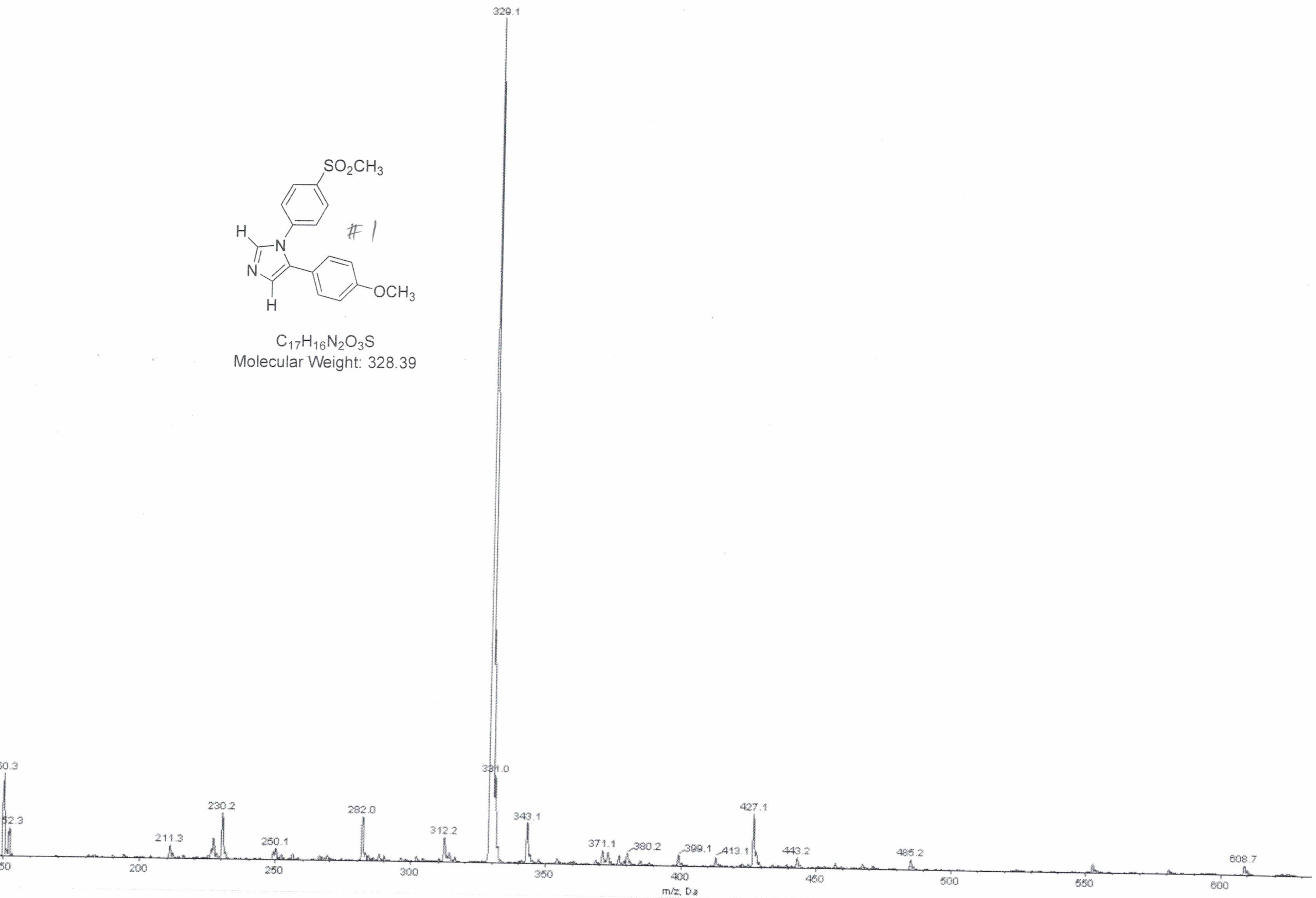

ple 1 (TuneSampleID) of Sample 9 (210709).wiff (Turbo Spray)

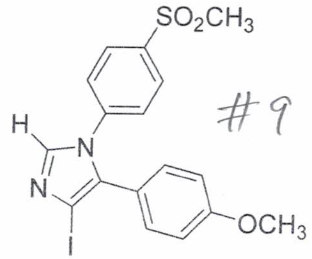

#9

$C_{17}H_{15}IN_2O_3S$
Molecular Weight: 454.28

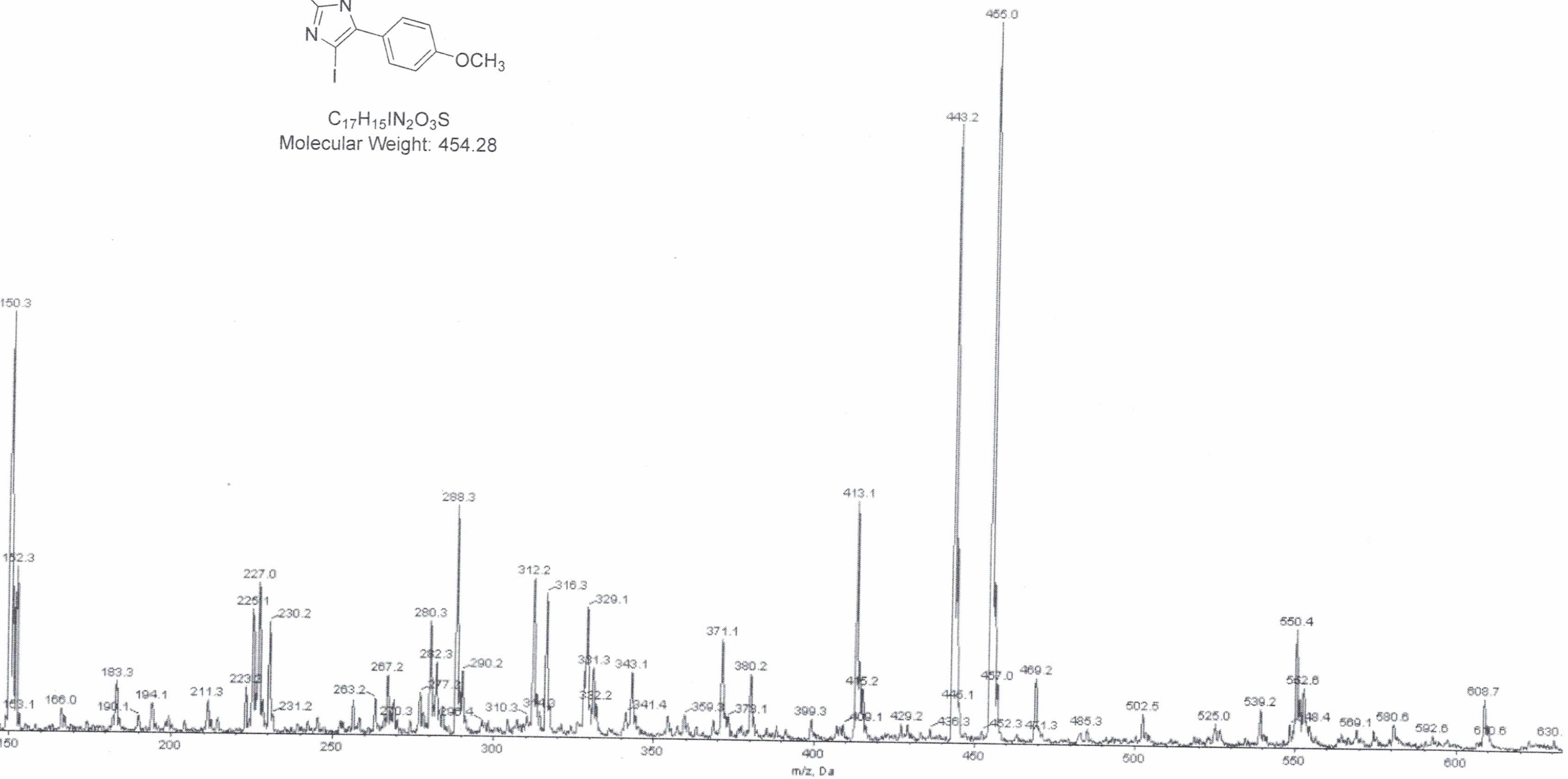

ile 1 (TuneSampleID) of Sample 10 (210709).wiff (Turbo Spray)

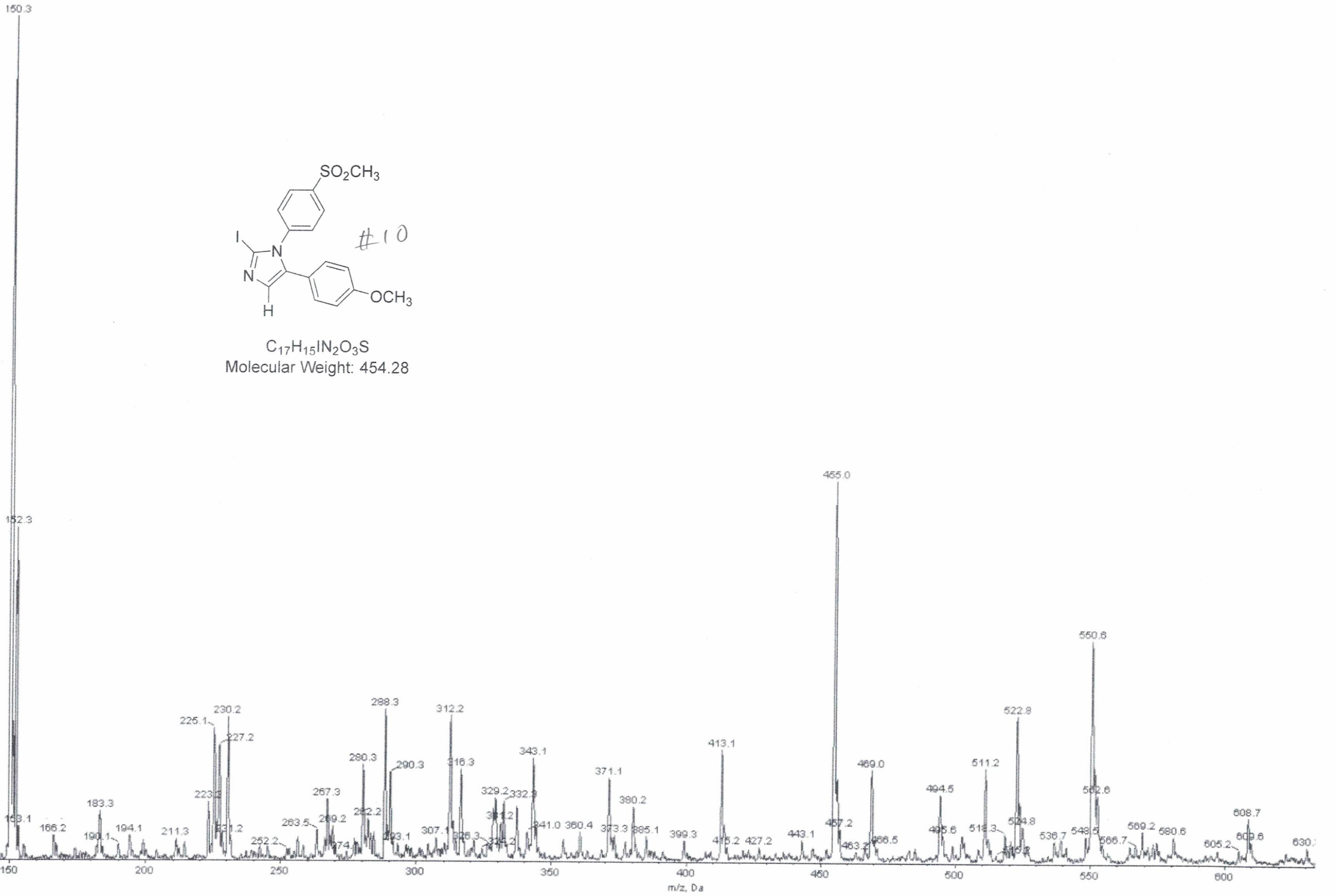

le 1 (TuneSampleID) of Sample 19 (210709).wiff (Turbo Spray)

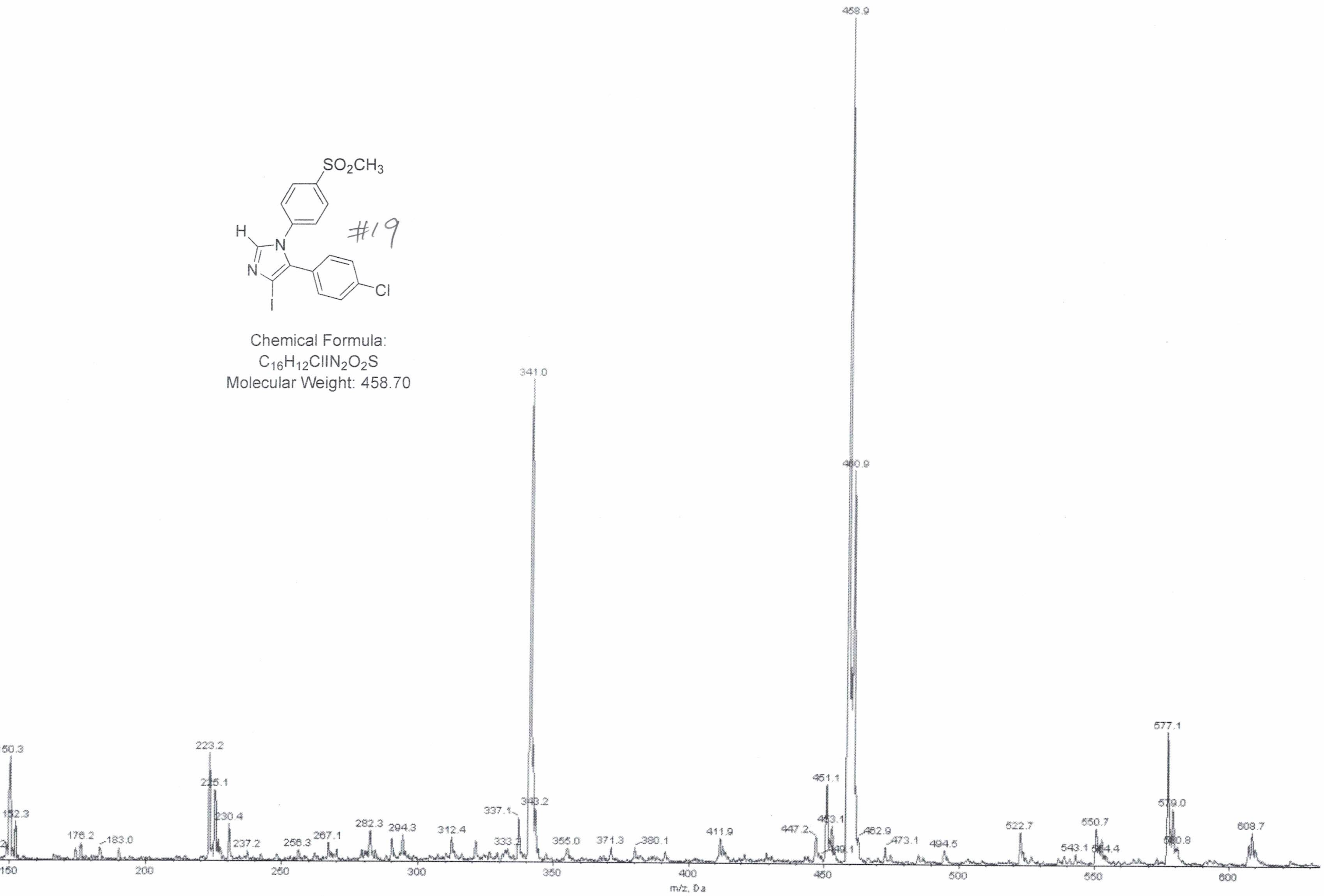

Chemical Formula:
$C_{16}H_{12}ClIN_2O_2S$
Molecular Weight: 458.70

HRMS data for 3, 7, 13, 16 (active compounds)

#3

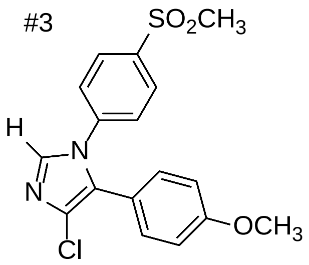

Chemical Formula: $C_{17}H_{15}ClN_2O_3S$
Exact Mass: 362.0492

Data : #3_re HR_2 Date : 07-Jul-2021 16:15
Instrument : MStation
Sample : -
Note : -
Inlet : Reservoir Ion Mode : EI+
RT : 0.77 min Scan# : 21
Elements : C 17/0, H 35/0, 35Cl 1/0, 37Cl 1/0, N 2/0, O 3/0, S 1/0
Mass Tolerance : 1mmu
Unsaturation (U.S.) : -0.5 - 20.0

	Observed m/z	Int%	Err[ppm / mmu]	U.S.	Composition
1	362.0493	100.00	+0.3 / +0.1	12.0	C17 H15 35Cl N2 O3 S

#7

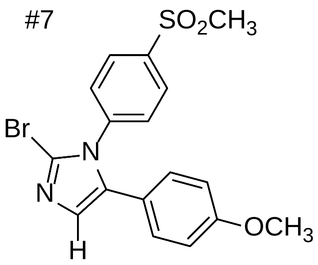

Chemical Formula: $C_{17}H_{15}BrN_2O_3S$
Exact Mass: 405.9987

Data : #7_re HR_3 Date : 07-Jul-2021 16:34
Instrument : MStation
Sample : -
Note : -
Inlet : Direct Ion Mode : EI+
RT : 0.35 min Scan# : 10
Elements : C 17/0, H 35/0, 79Br 1/0, 81Br 1/0, N 2/0, O 3/0, S 1/0
Mass Tolerance : 1mmu
Unsaturation (U.S.) : -0.5 - 20.0

	Observed m/z	Int%	Err[ppm / mmu]	U.S.	Composition
1	405.9988	100.00	+0.3 / +0.1	12.0	C17 H15 79Br N2 O3 S

#13

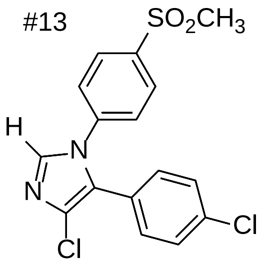

Chemical Formula: $C_{16}H_{12}Cl_2N_2O_2S$
Exact Mass: 365.9997

Data : #13_HR 3 Date : 07-Jul-2021 11:43
Instrument : MStation
Sample : -
Note : -
Inlet : Reservoir Ion Mode : EI+
RT : 0.73 min Scan# : 20
Elements : C 16/0, H 33/0, 35Cl 2/0, 37Cl 2/0, N 2/0, O 2/0, S 1/0
Mass Tolerance : 1mmu
Unsaturation (U.S.) : -0.5 - 20.0

	Observed m/z	Int%	Err[ppm / mmu]	U.S.	Composition
1	365.9994	100.00	-0.7 / -0.3	12.0	C16 H12 35Cl2 N2 O2 S

#16

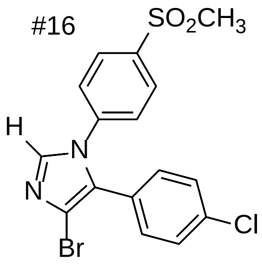

Chemical Formula: $C_{16}H_{12}BrClN_2O_2S$
Exact Mass: 409.9491

Data : #16_HR 5 Date : 07-Jul-2021 11:01
Instrument : MStation
Sample : -
Note : -
Inlet : Reservoir Ion Mode : EI+
RT : 0.88 min Scan# : 24
Elements : C 16/0, H 12/0, 79Br 1/0, 81Br 1/0, 35Cl 1/0, 37Cl 1/0, N 2/0, O 2/0, S 1/0
Mass Tolerance : 10mmu
Unsaturation (U.S.) : -0.5 - 100.0
Unsaturation (U.S.) : -0.5 - 100.0

	Observed m/z	Int%	Err[ppm / mmu]	U.S.	Composition
1	409.9490	100.00	-0.3 / -0.1	12.0	C16 H12 79Br 35Cl N2 O2 S
